# Investigation of Solid-State Thermal Decomposition of Ammonia Borane Mix with Sulphonated Poly(ellagic Acid) for Hydrogen Release

**DOI:** 10.3390/polym16243471

**Published:** 2024-12-12

**Authors:** Carmela Astorino, Eugenio De Nardo, Stefania Lettieri, Giuseppe Ferraro, Mattia Bartoli, Marco Etzi, Angelica Monica Chiodoni, Candido Fabrizio Pirri, Sergio Bocchini

**Affiliations:** 1 Center for Sustainable Future Technologies—CSFT@POLITO, Via Livorno 60, 10144 Torino, Italy; carmela.astorino@polito.it (C.A.); eugenio.denardo@polito.it (E.D.N.); giuseppe.ferraro@polito.it (G.F.); marco.etzi@iit.it (M.E.); angelica.chiodoni@iit.it (A.M.C.); fabrizio.pirri@polito.it (C.F.P.); sergio.bocchini@polito.it (S.B.); 2 Department of Applied Science and Technology, Politecnico di Torino, C.so Duca degli Abruzzi 24, 10129 Turin, Italy; 3 Consorzio Interuniversitario Nazionale per la Scienza e Tecnologia dei Materiali (INSTM), Via G. Giusti 9, 50121 Florence, Italy

**Keywords:** ammonia borane, hydrogen storage, bioderived polymers, ellagic acid

## Abstract

The utilization of hydrogen in safety conditions is crucial for the development of a hydrogen-based economy. Among all methodologies, solid-state hydrogen release from ammonia borane through thermal stimuli is very promising due to the high theoretical hydrogen release. Generally, carbonaceous or inorganic matrices have been used to tune the reactivity of ammonia borane. Nevertheless, these solutions lack chemical tunability, and they do not allow one to properly tune the complex chemical pathway of hydrogen release from ammonia borane. In this study, we investigated the effect of a bioderived multifunctional polymeric matrix on hydrogen release from ammonia borane, reaching pure hydrogen release of 1.2 wt.% at 94 °C. We also describe new chemical pathways involving the formation of anchored intermediates, namely BxNy species.

## 1. Introduction

The exploration of naturally derived building blocks in polymer synthesis is primarily driven by the pursuit of environmentally sustainable, biodegradable, and less toxic materials, with a focus on leveraging nature’s diversity for innovative and enhanced material properties, in response to both regulatory demands and consumer preferences for environmentally friendly products [1,2]. Their versatile properties, such as high mechanical strength, flexibility, and tunable chemical structures, make polymers an ideal platform for the development of innovative materials with a wide array of applications [3,4,5]. Polymers that blend favorable attributes obtained from aromatic rings (such as superior mechanical and thermal properties) with the potential for post-reactions or post-functionalization offer a wide range of opportunities for various applications [6,7]. Ellagic acid (EA) is a naturally occurring polyphenolic compound, abundantly found in several fruits and nuts, such as strawberries, pomegranates, and walnuts [8,9,10,11]. EA has attracted considerable attention in recent years, particularly for its remarkable antioxidant, anticancer, and anti-inflammatory properties, making it a promising candidate for biomedical applications [12,13,14]. It can be extracted from natural sources, although several studies have reported its synthesis [9] and functionalization even at a large scale [15,16]. The chemical structure of EA is unusual, comprising two gallic acid units connected by a central glucose molecule, resulting in a dimeric structure known as ellagitannin. The presence of multiple hydroxyl groups on the phenolic rings provides ample sites for chemical modification, facilitating the synthesis of various polymer architectures and enhancing the functionality of materials based on EA. Beyond food and biomedical applications, EA could represent a particularly interesting feedstock for the production of multifunctional polymers. To the best of our knowledge, a copolymer of a first-generation polymer of intrinsic microporosity (PIM-1) and EA, which represents the sole published instance of an EA-based polymer (PEA), has been reported for application in gas separation processes (specifically CO_2_/N_2_ and CO_2_/CH_4_) [17]. Furthermore, the use of such multifunctional microporous PEA is also very promising as a host material for the incorporation of solid-state hydrogen carriers such as ammonia borane (AB). AB is a hydrogen-dense carrier that has achieved theoretical hydrogen release of up to 19.6 wt.% [18]. Nevertheless, the solid-state chemical reactivity of AB involves several simultaneous reaction pathways [19], while AB hydrolysis is relatively easy to perform [20], together with other green routes, such as photocatalytic degradation, using several high-performing catalysts [21,22,23,24,25].

The release of hydrogen-rich intermediates such as borazine or AB dimers and the regenerability of solid-state systems based on AB are the main issues to be addressed [26,27]. Entrapping the volatile intermediates and the successive reactions between them represents a very interesting approach to modulate hydrogen release from AB under thermal stimuli [28]. Commonly, AB is trapped in carbonaceous [29,30] or inorganic porous materials [31,32] to avoid this setback. Nonetheless, the common confinement routes lack in tunability of the matrix’s chemical features. In the present work, we propose a new system based on an AB blend with sulfonated poly(ellagic acid) (SPEA) as a reliable tool to tune the reactivity of AB using a porous functional polymeric matrix produced from biologically abundant resources. We herein report a detailed study on the interactions between AB and SPEA by monitoring the thermal degradation using thermogravimetric analysis coupled with IR (TGA-IR), providing new insights into the system’s reactivity.

## 2. Materials and Methods

### 2.1. Materials

All solvents and reagents were used as received, without further purification. EA was purchased from Biosynth (Siena, Italy). All other reagents and solvents were purchased from Merck (Sigma-Aldrich, St. Louis, MO, USA).

### 2.2. Synthesis of SPEA, AB, and AB@SPEA

The reaction was carried out according to the scheme reported in the Supporting Information (Figure S1). A mixture of anhydrous K_2_CO_3_ (4.5 eq, 5.53 g), EA (1 eq, 2.66 g), and 2,3,5,6-tetrafluoroterephthalonitrile (1 eq, 1.76 g) in dry N-methylpyrrolidone (30 mL) was stirred at 65 °C for 24 h under N_2_, adapting the procedure reported by Hossain et al. [17]. A whitish precipitate was formed and then washed several times with fresh methanol. The as-obtained PEA was dried overnight under a vacuum. Then, 1.2 g of PEA was stirred overnight at room temperature (RT) in 20 mL H_2_SO_4_. The as-obtained SPEA was then precipitated in distilled water and washed several times until a neutral pH in the washing water was obtained.

AB was produced as reported by Ramachandran et al. [33]; briefly, sodium borohydride (1 eq, 3.78 g) and ammonium sulfate (1 eq, 13.21 g) were mixed at 40 °C in tetrahydrofuran for 3 h. The reaction mixture was filtered and the recovered solution was dried under a vacuum to obtain AB. The solid was further dried at 40 °C under a vacuum for 16 h.

AB@SPEA mixtures were prepared by dissolving 50 mg of SPEA+AB in tetrahydrofuran. Two different weight ratios of AB:SPEA were investigated: 5:1 (AB@SPEA 5) and 1:1 (AB@SPEA 1). The solutions were dried at room temperature and then under a vacuum at 40 °C overnight before further analysis.

### 2.3. Physicochemical Characterization of SPEA and AB@SPEA

TGA-IR analyses were carried out using a thermogravimetric analyzer, the NETZSCH TG 209 F1, connected by a transfer line heated at 230 °C with an IR Bruker TENSOR II equipped with an IR gas cell heated at 200 °C. For AB@SPEA, the tests were performed by heating ~3 mg of the sample in alumina pans from 30 to 350 °C with a rate of 10 °C min^−1^ under a N_2_ flow of 40 mL min^−1^. For PEA and SPEA, ~10 mg of each sample was analyzed in a temperature range of 30 to 800 °C with a rate of 10 °C min^−1^ under a N_2_ flow of 40 mL min^−1^.

Fourier-transformed IR in attenuated total reflection (FT-IR ATR mode) was employed to characterize the membranes. Measurements were carried out on a Bruker Tensor II Fourier transform spectrophotometer. The spectra were acquired by accumulating 64 scans (64 for the background spectrum) in the 500–4000 cm^−1^ range with a resolution of 2 cm^−1^.

Differential scanning calorimetry (DSC) was performed using a Netzsch DSC 204 F1 Phoenix instrument, equipped with a low-temperature probe. The experiments were carried out between −70 and 180 °C in a N_2_ atmosphere (70 mL min^−1^) by weighing 3 mg of each sample. The activation energy of the dehydrogenation process of AB@SPEA was calculated by using the Kissinger equation with data collected through DSC analyses using heating rates of 2, 5, and 10 °C min^−1^.

XRD analyses were performed using a Panalytical X’PERT PRO PW3040/60 diffractometer (Cu K α radiation at 40 kV and 40 mA, Panalytical BV, Almelo, The Netherlands). The diffraction spectra were obtained from powder in the 2θ range from 15 to 60° with a step size of 0.013°. The XRD spectra were analyzed using the freeware QualX software 2.0.

## 3. Results

### 3.1. Evaluation of Dehydrogenation Activation Energy of AB@SPEAx (x = 1, 5)

**Figure 1:**
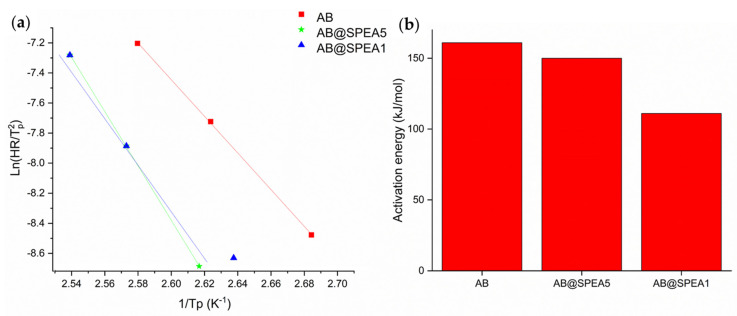
Data output of DSC elaboration using Kissinger equation reported as (**a**) Kissinger plot and (**b**) activation energies of AB@SPEAx (x: 1 or 5) during first dehydrogenation.

The solid-state reactivity of AB is rather complex [18] due to the involvement of several degradative pathways, including the release of ammonia, boron-based species, and pure hydrogen. The release of molecular hydrogen occurs at the very early stage of AB’s thermal decomposition, and it is easily visible through the DSC analysis of AB-containing samples as an exothermic peak at 90 to 110 °C, depending on the surrounding chemical environment [34,35]. The exothermic peak related to the first dehydrogenative step of AB can be used for the evaluation of the activation energy (E_a_) using the non-isothermal Kissinger equation [36]:

(1) $$\ln\left(\frac{HR}{T_{p}^{2}}\right)=\frac{{-E}_{a}}{{RT}_{p}}+\ln\left(\frac{Z}{{RE}_{a}}\right)$$

where R is the ideal gas constant (8.31 J mol^−1^ K^−1^), HR is the heating rate used (2, 5, 10 °C min^−1^), *T*_p_ is the exothermic peak temperature in K as reported in the Supporting Information (Figure S4), E_a_ is the activation energy of the dehydrogenative process, and Z is a kinetic constant. E_a_ was evaluated by plotting ln(*α*/*T*_p_^2^) versus 1/*T*_p_ using a linear fit, as shown in Figure 1.

As reported in Figure 1a, the Kissinger plots of AB@SPEAx (x = 1, 5) showed significant differences compared with pure AB, suggesting a relevant improvement in the kinetics of the thermal degradative process. AB dehydrogenation showed an E_a_ of up to 168 kJ mol^−1^, in agreement with the previous work by Gutowska et al. [37] (E_a_ = 161 kJ mol^−1^). The E_a_ of AB@SPEA5 showed a value of 148 kJ mol^−1^, while the E_a_ of AB@SPEA1 was 112 kJ mol^−1^. The trend of the E_a_ suggests a direct correlation between the decrease in the AB/SPEA ratio and AB destabilization. This is reasonable due to the destabilization of the N-B bond from the interaction with the sulfuric residues present on the SPEA chains, in agreement with the previous findings of Chandra et al. [38] regarding sulfonic acid resins.

### 3.2. Thermal Degradative Process of AB@SPEAx (x = 1, 5)

**Figure 2:**
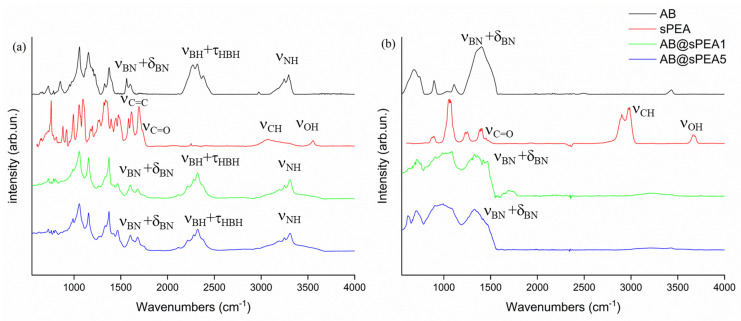
FT-IR spectra (ATR mode) of AB (black line), SPEA (red line), AB@SPEA1 (blue line), and AB@SPEA5 (green line) (**a**) pre- and (**b**) post-TGA-IR analysis in the range between 500 and 4000 cm^−1^. The more relevant peaks are highlighted.

The FT-IR SPEA spectra of all samples are reported in Figure 2. The FT-IR spectrum of AB showed a broad band composed of three components centered at three large and intense peaks at around 3178, 3247, and 3298 cm^−1^ due to ν_NH_, while symmetric and asymmetric ν_BH_ were centered at 2382, 2316, and 2270 cm^−1^ [39]. As mentioned by Hess et al. [40], there were other two bands centered at 2426 and 2212 cm^−1^, which cannot be attributed to a well-defined atomic vibration but to a composition of lattice vibrations. The umbrella and scissoring modes of -NH_3_ occurred at 1603 and 1327 cm^−1^, while the ones related to -BH_3_ were detected at 1186 and 1160 cm^−1^, respectively. A split band at 1380 and 1323 cm^−1^ was due to the overtones of -NH_3_ deformations, as previously described [40]. ρ_BNH_ was observed at 1052 cm^−1^ while the symmetric and asymmetric ν_BN_ were observed at 843 and 717 cm^−1^, respectively. The spectrum of SPEA showed a characteristic ν_OH_ peak at 3554 cm^−1^ due to the hydroxylic terminations [41] and a broad band centered at 3077 cm^−1^ due to unsaturated ν_CH_. The polymerization was proven by the presence of a sharp peak centered at 2245 cm^−1^ due to the nitrile ν_CN_, while the symmetrical and asymmetrical ν_S = O_ bands were centered at 1184 and 1171 cm^−1^, respectively, proving the sulfonation of poly(ellagic acid). The AB@SPEAx(x = 1, 5) showed the same band pattern as AB with shoulders and the broadening of the peaks due to the presence of SPEA.

**Figure 3:**
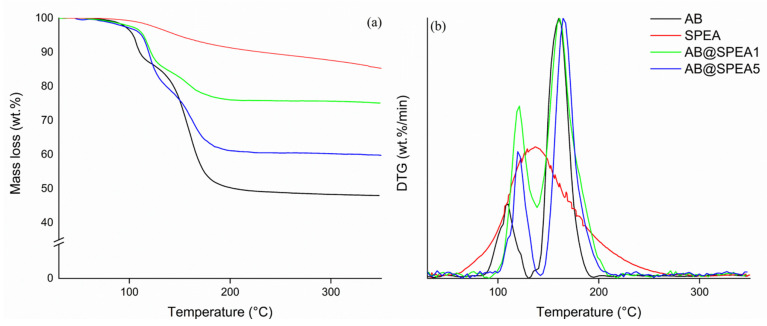
TGA (**a**) and DTG (**b**) of AB (black line), SPEA (red line), AB@SPEA1 (blue line), and AB@SPEA5 (green line).

**Figure 4:**
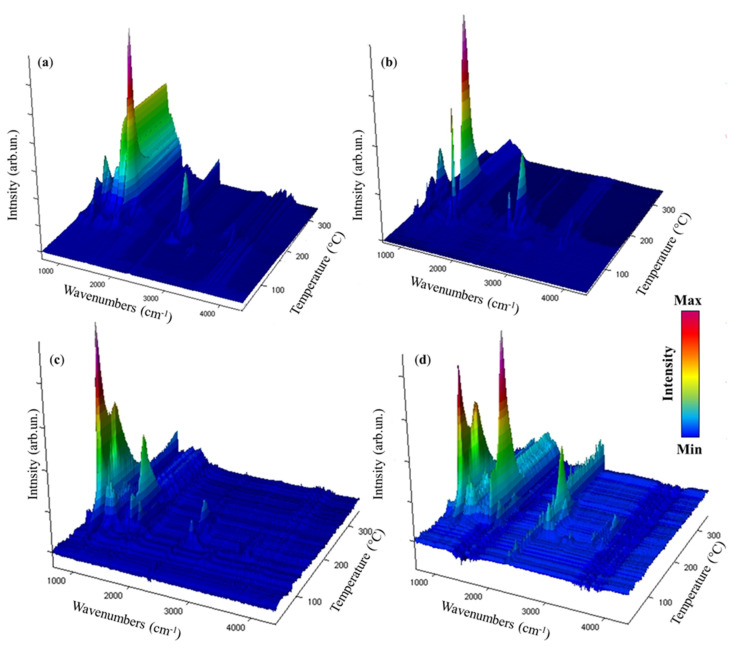
IR spectra collected during the thermal degradation of (**a**) AB, (**b**) SPEA, (**c**) AB@SPEA1, and (**d**) AB@SPEA5 in the temperature range of 30–350 °C.

A detailed degradative investigation of AB, SPEA, and AB@SPEAx(x = 1, 5) was conducted using TGA-IR with quantitative analysis based on the thermograms and FT-IR spectra reported in Figure 3 and Figure 4, respectively, and summarized in Table 1.

The quantification of SPEA functionalization was challenging due to its poor solubility in any solvent. Nevertheless, the TGA-IR analysis of SPEA can be compared to the one run with the PEA precursors in order to evaluate the effectiveness of the sulfonation process (see Supporting Information, Figures S2 and S3). The molecular weight of SPEA was 588.44 g mol^−1^, and the sulfonic residues that represented 27.6 wt.% of SPEA showed a weight loss of up to 16.8% in the range of up to 400 °C (see Supporting Information, Figure S3), while PEA lost up to 6.4 wt.%. As shown by the FT-IR spectra of the gas released during the main degradative stages of the TGA-IR analysis (see Supporting Information, Figure S4), SPEA and PEA released CO_2_ and CO as a consequence of ester function degradation; moreover, SPEA released also SO_2_ due to the degradation of the sulfonic residues. Accordingly, the degree of sulfonation, calculated as (mass loss of SPEA-mass loss of PEA) in the temperature range of up to 400 °C, was up to 37.7%.

AB and AB@SPEAx (x = 1, 5) showed major degradative steps that included several simultaneous reaction pathways, as shown in Figure 4.

According to Figure 4a, AB released molecular H_2_ at up to 61 °C, with a mass loss of up to 0.2 wt.%, while the release of both NH_3_ and B_2_H_6_ was confirmed by the new IR feature due to ν_NH_ at 965 cm^−1^ and 930 cm^−1^ [42] and diborane τ_HBH_ at 2584–2523 cm^−1^, ν_BH_ at 2361–2329 cm^−1^, and δ_BH_ at 1387 cm^−1^ [43]. The presence of these species suggested an initial degradative pathway involving the cleavage of the N-B bond with the coupling of two molecules of BH_3_ in the gas phase [44]. At 86 °C, we observed bands of ν_BN_ of NH_2_BH_2_ centered at 1392 cm^−1^ and 1334 cm^−1^ [45], while, at temperatures higher than 100 °C, borazine was formed, producing typical bands at 1470–1460 cm^−1^ and 2741–2396 cm^−1^, as described by Kaldor et al. [46]. The borazine formation reached the maximum at 162 °C, with a residue at 350 °C of up to 48.0 wt.%. AB@SPEA5 showed the release of molecular H_2_ at up to 83 °C, with the gravimetric release of 0.9 wt.%. Interestingly, AB@SPEA5 released a mixture of NH_3_ and B_2_H_6_ up to 123 °C, accordingly with the mechanism of reaction described by Al-Kukhun et al. [47]. From 123 °C onward, AB@SPEA5 released dimers of AB until 207 °C, when it released dehydrogenated borazine and NH_3_. Similarly, AB@SPEA1 released pure H_2_ up to 94 °C, with gravimetric release of up to 1.2 wt.%. In this case, only NH_3_ was released up to 111 °C, when a degradative pathway activated the release of B_2_H_6_, while dehydrogenated borazine and NH_3_ were released from 207 °C.

### 3.3. Mechanistic Insights into the Thermal Degradative Process of AB@SPEAx (x = 1, 5)

**Figure 5:**
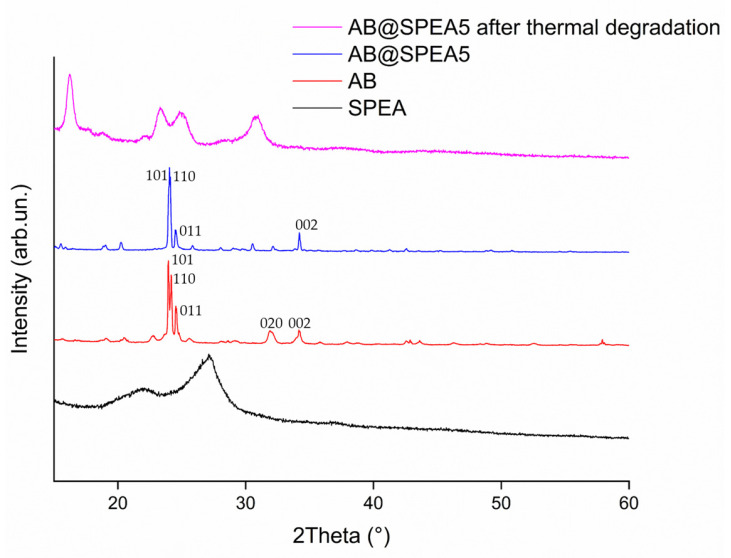
XRD spectra of AB, SPEA, AB@SPEA5, and AB@SPEA5 after thermal degradation.

As shown in Figure 2b, the solid residues of AB@SPEAx (x = 1, 5) were considerably different compared with AB, showing a broader envelope of bands between 1565 and 1190 cm^−1^ due to ν_BN_ and δ_BN_; in addition, we observed, for AB@SPEA, the envelopment of the bands between 1000 and 550 cm^−1^ due to skeletal modes that were closer to the one observed for boron carbon nitride [48]. As shown in Figure 5, SPEA showed broad signals centered at 21.8° and 27.1°, while AB showed a signal pattern with peaks centered at 23.9°, 24.2°, 24.5°, 31.9°, and 34.2°, suggesting the presence of an orthorhombic phase, as reported by Hoon et al. [49]. AB@SPEA5 showed a similar pattern in which peak 020 shifted at 32.1°, with a considerable reduction in intensity, suggesting the presence of a tetragonal phase, while the broad signal of SPEA was not appreciable. This was reasonable due to the temperature treatment applied to remove the solvent used during the SPEA mixing. Interestingly, AB@SPEA5, after thermal treatment, showed XRD spectra close to the one of AB treated at more than 1500 °C, as reported by Frueh et al. [50], with four peaks centered at 16.3°, 23.3°, 29.9°, and 30.9°. This suggests the low-temperature rearrangement of the N_x_B_y_ species formed at 350 °C during the degradation of AB.

**Figure 6:**
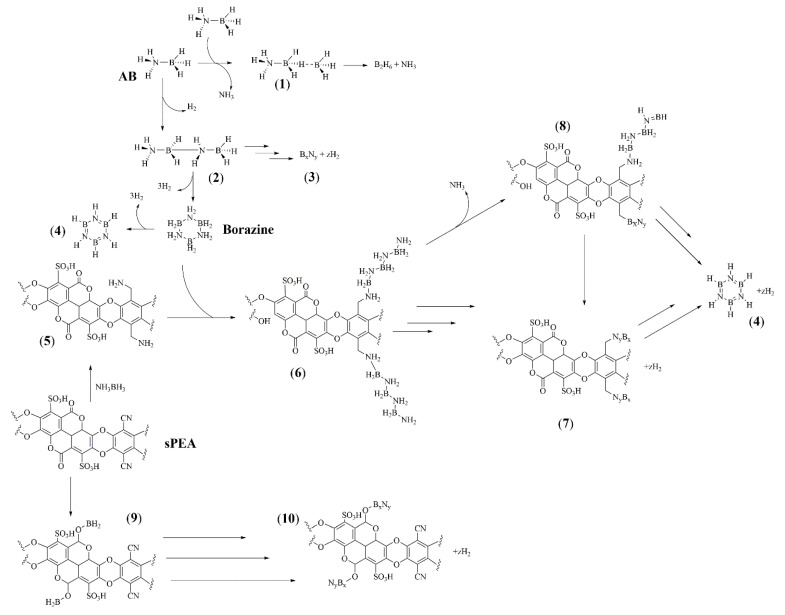
Hypothetical chemical pathways occurring during the thermal degradation of AB@SPEAx (x = 1, 5) with intermediated numbered from **1** to **10**.

Accordingly, we hypothesized a mechanism of degradation for AB in the presence of SPEA, as reported in Figure 6.

**Figure 7:**
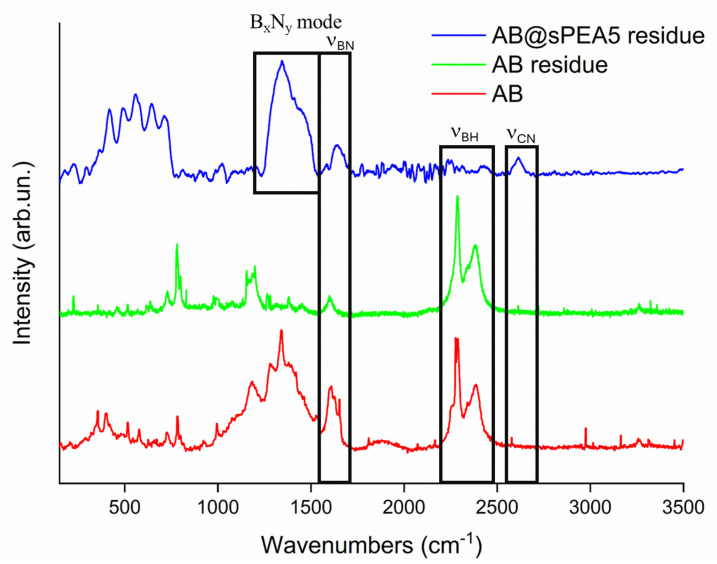
Raman spectra of AB, AB residue, and AB@SPEA5 in the range of 250 to 3500 cm^−1^.

AB undergoes a complex degradative mechanism, moving through the formation of **1** and linear dehydrogenated B_x_N_y_ (**3**) or through 2 with the release of borazine and a mixture of **4** and H_2_. Nevertheless, SPEA displayed several interesting reactive residues, ranging from nitriles to esters in the presence of sulfonated residues, which can act as acid catalyst sites. The reactivity of AB with these species is well known [51] and it can form intermediates such as **5** and **9** [52]. As reported by Bartoli et al. [29], borazine is generally released at temperatures higher than 100 °C, but we solely observed **4** at temperatures higher than 200 °C. This suggests that the occurrence of the reaction between borazine and reduced SPEA derivatives in the presence of acid catalysis is promoted by sulfonic residues, forming species such as **6** and **10** through nucleophilic addition reactions [53]. Furthermore, the mechanism of formation, such as the one leading to the production of **8**, explains the formation of NH_3_ across the overall degradative process, with the N-terminal of anchored B_x_N_y_ being released, reducing the formation of linear dehydrogenated species. This agrees with the IR of the residues that showed similarity with carbon boron nitride and with the release of dehydrogenated borazine. As shown in Figure 7, the Raman analysis showed the advancement of the dehydrogenation of AB with the disappearance of the ν_BN_, while AB@SPEA5 showed the complex envelopment of the bands between 1500 and 1600 cm^−1^. Furthermore, the disappearance of the ν_CN_ supports the mechanism reported in Figure 5, with the anchoring steps of B_x_N_y_ species.

This latter species was released at temperatures higher than 200 °C, suggesting a trade-off between the length of B_x_N_y_ and their stability.

## 4. Conclusions

Confinement in a specialized matrix is a well-established practice to provide a solid tool for the controlled thermal degradation of AB. In this study, we synthesized a bioderived multifunctional polymeric matrix that promoted the solid-state reaction pathways of AB by fine-tuning the AB/SPEA interaction. Accordingly, we reported hydrogen storage release up to 1.2 wt.% and the modulation of the E_a_ with a decrement of up to 31%. The reduction in the E_a_ is of particular interest due to the possibility of hydrogen release at lower temperatures, coupled with a system that is able to entrap the eventual side products produced. The hypothesized mechanism, together with the TGA-IR and FT-IR outputs, clearly shows the entrapping of volatile species on specific sites of SPEA, increasing the hydrogen mixed with BxNy dehydrogenated species. AB@SPEAx (x = 1, 5) represents the first successful attempt to modulate the AB solid-state reactivity by using a polymeric matrix that is able to entrap the intermediates formed with a reduction in temperature release.

## Figures and Tables

**Table 1:** Results from TGA analyses in nitrogen for AB, SPEA, and AB@SPEAx(x = 1, 5).

Sample	T_onset_ (°C)	T_max1_ (°C)	Residue @ T_max1_ (%)	T_max2_ (°C)	Residue @ T_max2_ (%)	Residue @ 350 °C (%)	H_2_ Solo Production (wt.%) ^a^
AB	105	106	93.4	162	75.1	48.0	0.23
SPEA	97	144	91.2	---	---	14.7	---
AB@SPEA1	82	118	84.2	158	75.4	75.2	1.2
AB@SPEA5	86	119	78.9	164	62.1	60.0	0.9

^a^ Calculated as (mass loss of AB@SPEAx(x = 1, 5) prior to the release of any IR detectable compound)-(mass loss of SPEA in the same temperature range).

## Data Availability

The original contributions presented in the study are included in the article/Supplementary Materials, further inquiries can be directed to the corresponding author/s.

## Supplementary Materials

The following supporting information can be downloaded at: https://www.mdpi.com/article/10.3390/polym16243471/s1, Figure S1: (a) DSC analysis of AB, (b) AB@sPEA5, and (c) AB@sPEA1 using 2, 5, and 10 °C/min as the heating rate, Figure S2: Chemical pathway for the production of SPEA using EA and 2,3,5,6-tetrafluoroterephthalonitrile as precursors, Figure S3: Thermograms of PEA and SPEA in the range of up to 800 °C in a nitrogen atmosphere; Figure S4: FT-IR spectra of gas released during the main degradative stages of PEA and sPEA in a nitrogen atmosphere during TGA-IR analysis.
