# Supplementary material for: Investigation of Solid-State Thermal Decomposition of Ammonia Borane Mix with Sulphonated Poly(ellagic Acid) for Hydrogen Release
Source: Polymers (Basel). 2024 Dec 12;16(24):3471. doi: 10.3390/polym16243471 (PMC11728564; doi:10.3390/polym16243471)

Figure S1: DSC analysis of a) AB, b) AB@sPEA5 and c) AB@sPEA1 using 2, 5 and 10 °C/min as heating rate.

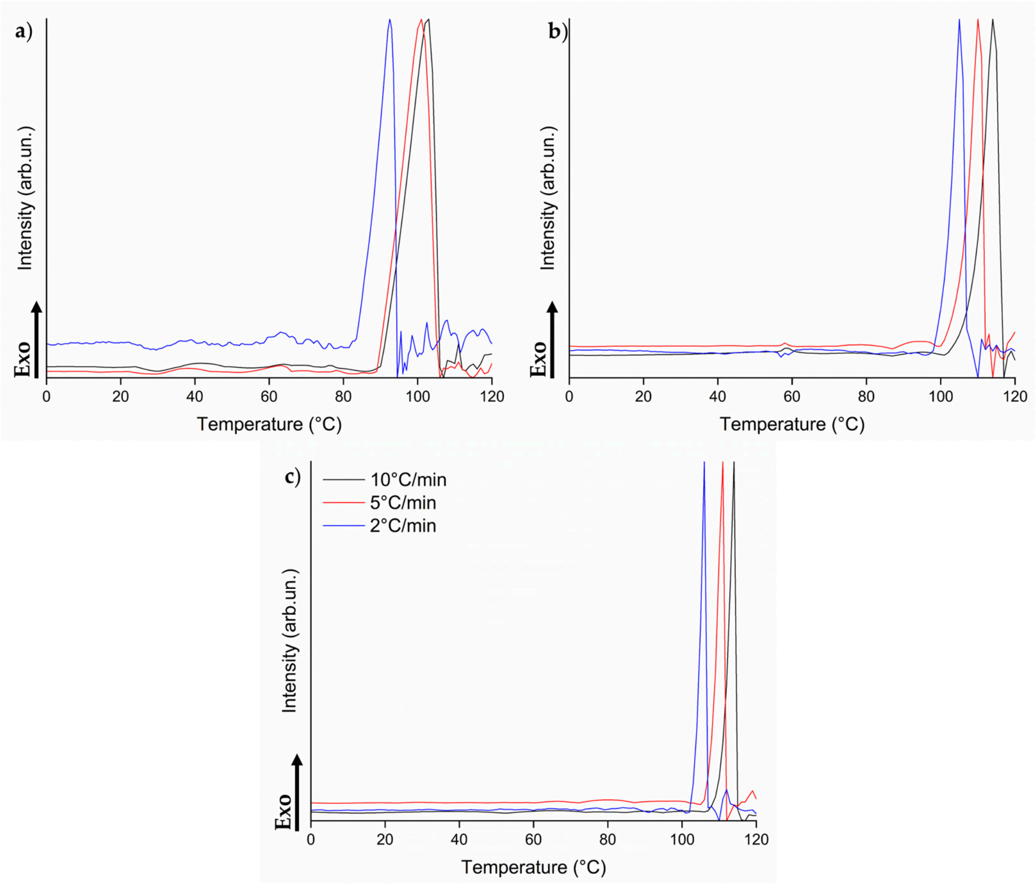

Figure S2: Chemical pathway for the production of sPEA using EA and 2,3,5,6-Tetrafluoroterephthalonitrile as precursors.

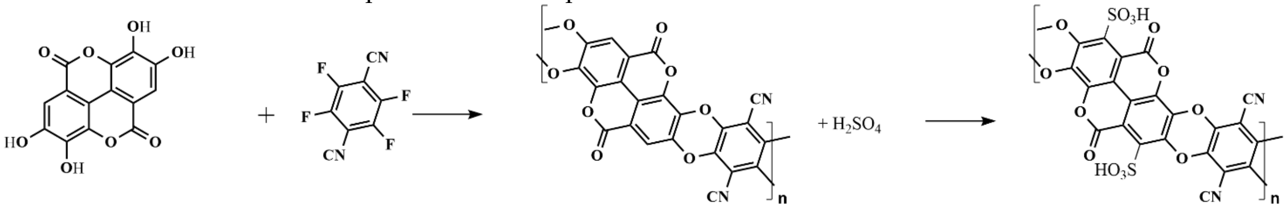

Figure S3: Thermograms of PEA and sPEA in the range up to 800°C in nitrogen atmosphere. TG curves are reported in black while DTG are reported in blue.

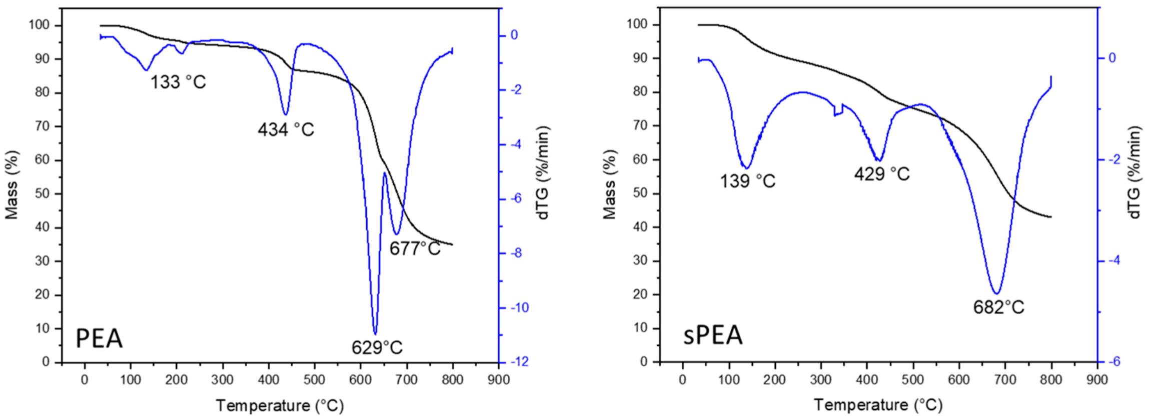

Figure S4: FT-IR spectra of gas released during the main degradative stages of PEA and sPEA in nitrogen atmosphere during TGA-IR analysis.

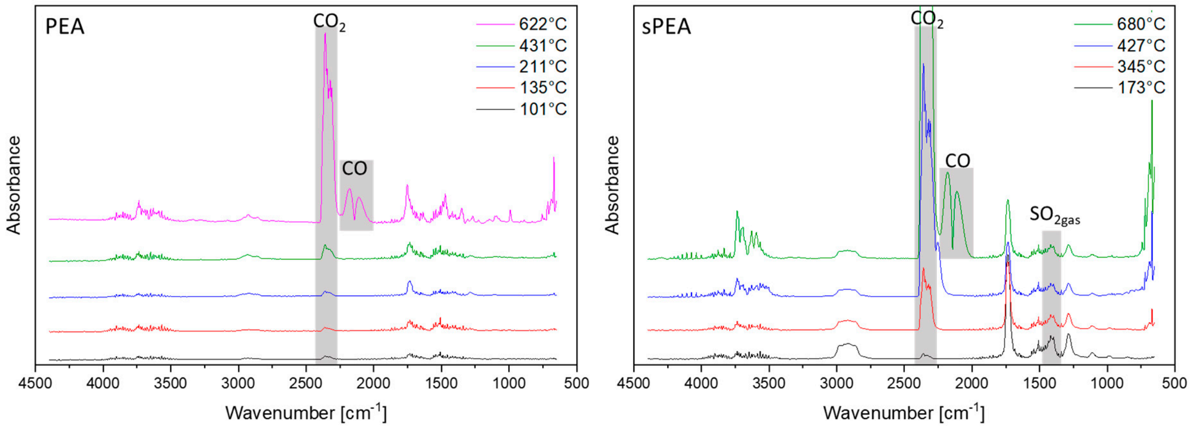

Supplement: Supplementary file 1 [file polymers-16-03471-s001.zip › polymers-3298293-supplementary.pdf]
